# Mitochondrial donation in translational medicine; from imagination to reality

**DOI:** 10.1186/s12967-020-02529-z

**Published:** 2020-09-25

**Authors:** Hesam Saghaei Bagheri, Farhad Bani, Savas Tasoglu, Amir Zarebkohan, Reza Rahbarghazi, Emel Sokullu

**Affiliations:** 1 School of Medicine, Biophysics Department, Koç University, Rumeli Fener, Sarıyer, Istanbul, Turkey; 2 Department of Medical Nanotechnology, Faculty of Advanced Medical Sciences, Tabriz University of Medical Sciences, Tabriz, Iran; 3 Koç University Translational Medicine Research Center (KUTTAM) Rumeli Feneri, Sarıyer, Istanbul, Turkey; 4 Faculty of Engineering, Mechanical Engineering Department, Koç University, Rumeli Feneri Yolu, Sarıyer, Istanbul, Turkey; 5 Stem Cell Research Center, Tabriz University of Medical Sciences, Tabriz, Iran; 6 Department of Applied Cell Sciences, Faculty of Advanced Medical Sciences, Tabriz University of Medical Sciences, Imam Reza St., Daneshgah St., 51666-14756 Tabriz, Iran

**Keywords:** Stem cells, Mitochondrial transfer, Cellular mechanisms, Regenerative potential

## Abstract

The existence of active crosstalk between cells in a paracrine and juxtacrine manner dictates specific activity under physiological and pathological conditions. Upon juxtacrine interaction between the cells, various types of signaling molecules and organelles are regularly transmitted in response to changes in the microenvironment. To date, it has been well-established that numerous parallel cellular mechanisms participate in the mitochondrial transfer to modulate metabolic needs in the target cells. Since the conception of stem cells activity in the restoration of tissues’ function, it has been elucidated that these cells possess a unique capacity to deliver the mitochondrial package to the juxtaposed cells. The existence of mitochondrial donation potentiates the capacity of modulation in the distinct cells to achieve better therapeutic effects. This review article aims to scrutinize the current knowledge regarding the stem cell’s mitochondrial transfer capacity and their regenerative potential.

## Background

The discovery of stem cells has revolutionized human medicine and these cells are touted as a promising therapeutic modality in the alleviation of different injuries [1]. During past decades, several clinical trials using multiple stem cells, especially MSCs, provide alternative therapeutic approaches to replace injured cells with the functional cells [2]. Compared to other stem cells, MSCs are easily accessible from mesenchymal tissues and could be produced in large scales for point-of-care delivery [3]. Meanwhile, the bio-preservation of MSCs has minimal potency loss and karyotypic abnormalities compared to adult somatic cells and other stem cell types [4]. Due to the lack of comprehensive understanding related to MSCs mechanism of action, further investigations are highly demanded to address the quantity and quality of therapeutic effects driven by MSCs after transplantation into the target sites. Up to date, it has been shown that these cells accelerate the repair of injured areas via engaging different underlying activities such as differentiation into distinct cell lineage, the release of arrays of cytokines and growth factors, and the promotion of cell-to-cell connection [5]. Rapid advances in the field of stem cell biology help us to discover very special restorative approaches used by stem cells to return the function of target cells after exposure to the insulting agents. In this regard, it has been well-documented that the transfer of mitochondrial from MSCs to acceptor cells is an important endogenous regeneration mechanism in the context of tissue regeneration [6]. Despite significant signs of progress in the elucidation of mitochondrial donation, the distinct mechanisms and critical factors involved in this process have not been fully discovered. Here, in this review article, we focused on the potency of stem cells, especially MSCs, to donate mitochondria to the non-stem cells under pathological conditions. In addition to an important role of mitochondria in stem cell differentiation, different intracellular machinery involved in the mitochondrial donation were also reviewed. It seems that these data will help us to understand the potency of stem cells, mainly MSCs, in the restoration of injured tissues via mitochondrial donation.

## The role of mitochondria in cell metabolism

Metabolism is the complex of vital chemical reactions inside single organisms [7]. This process includes food conversion to energy in response to the energy demands by synthesizing blocks for proteins, lipids, nucleic acids, and carbohydrates; and the elimination of nitrogenous wastes. With the promotion of cell metabolism, various intermediate factors are interchanged between the cells in a tightly regulated manner [8]. Both catabolic and anabolic metabolic reactions are tightly regulated under physiological conditions. As a common belief, different forms of cellular metabolism depend on redox reactions that involve the transfer of electrons from reduced donor molecules such as organic molecules, water, ammonia, hydrogen sulfide or ferrous ions to acceptor molecules such as oxygen, nitrate or sulfate [9]. In this regard, the mitochondrion, as a major source of cellular energy, has a key role in the promotion of catabolic and anabolic reactions. Interestingly, the number of mitochondria differs based on cell type and cell growth stage [10]. For instance, erythrocytes are devoid of mitochondria, whereas hepatocytes have more than 2000 mitochondria per each cell [11].

### Mitochondrion ultrastructure

The mitochondrion contains outer and inner membranes composed of phospholipid bilayers and proteins. The outer membrane, encompassing the entire mitochondrial space, has a thickness of 60–75 Å. The protein/phospholipid ratio in the outer membrane is similar to that of the eukaryotic cell membrane. A large number of integral membrane proteins, termed porins, are present in the outer membrane. An inter-membrane space exists between the outer and inner layers, which is also termed peri-mitochondrial space. Due to the permeability of the outer membrane to small-size biomolecules, the concentration of ions and small polysaccharides in both sides of the outer membrane is the same. There are functional proteins in the inner membrane that make this layer eligible for cellular bioactivity. According to previously published data [12, 13], several functional proteins are integrated into the inner membrane. These proteins participate in the redox reaction chain of oxidative phosphorylation; synthesis of ATP in the mitochondrial matrix; transportation of specific molecules inside and outside of mitochondrial matrix; and mitochondrial fusion and fission.

### Mitochondrial metabolism

**Fig. 1:**
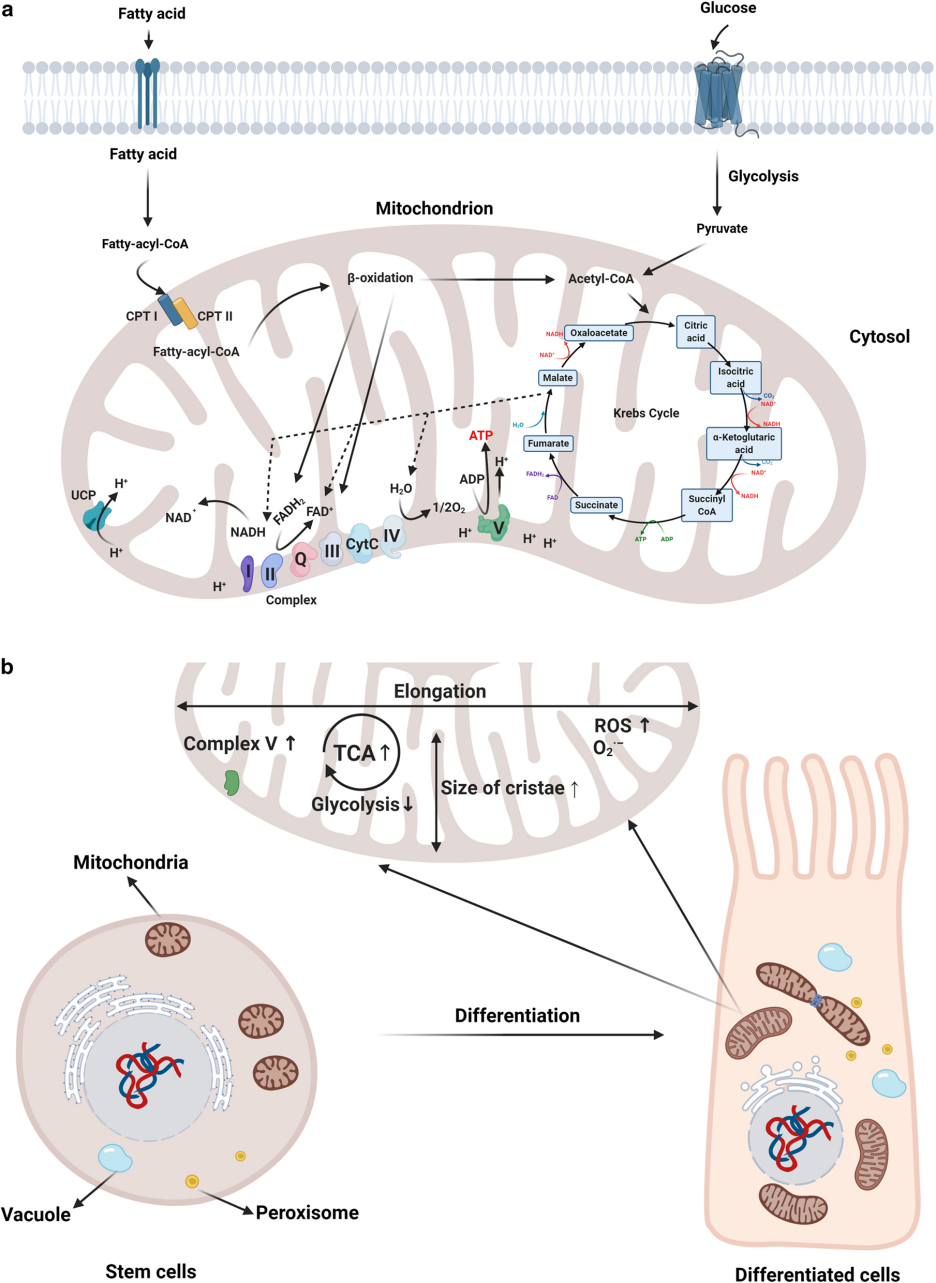
Mitochondria act as a powerhouse of the cell (**a**). Metabolic pathways within mitochondria contribute to molecular biosynthesis and the production of ATP. Inside mitochondrial, pyruvate, fatty acids, and amino acids were oxidized and electrons enter to electron transport chain. The production of ATP is facilitated by an electrochemical gradient through oxidative phosphorylation. The role of mitochondria during differentiation (**b**).The size and number of mitochondria are increased in stem cells along with maturation to the mature cell types. In the progress of differentiation, mitochondria are elongated and the length of crista increase because of active oxidative phosphorylation. It seems that reactive oxygen species and oxygen radicals increase by activation of mitochondrial function. Carnitine palmitoyltransferase I, II: CPT I and II; Cytochrome c: CytC; Flavin adenine dinucleotide: FADH; Nicotinamide adenine dinucleotide: NADH; Reactive oxygen species: ROS; Tricarboxylic acid: TCA; Uncoupling proteins: UCPs

Oxidative phosphorylation is touted as the main biochemical reaction required for the production of cellular energy and is considered as a powerhouse. Oxidative phosphorylation is governed by coordinated cascades of redox reactions via protein complexes located in the inner mitochondrial membrane, which is known as the electron transport chain complex (Fig. 1a). Inside the mitochondrial matrix, enzymes related to the tricarboxylic acid (ETC) cycle provide electron transporters such as nicotinamide adenine dinucleotide (NADH) and flavin adenine dinucleotide (FADH_2_) [14]. This collection consists of I-IV protein complexes, soluble factors, cytochrome C oxidase, and coenzyme Q which deliver electrons to the ETC.

Complex I and II transfer two electrons from the electron carriers (NADH/FADH2) to the coenzyme Q. Complex III is an adaptor effector that uptakes two electrons from the reduced form of coenzyme Q and subsequently transfers electrons to cytochrome C oxidase (Fig. 1a). Complex IV interrupts the respiratory chain after the transfer of electrons from cytochrome C oxidase by the reduction of oxygen to produce water [15, 16]. The unique reduction/oxidation reactions contribute to conformational changes in ETC of respiratory complexes, enabling mitochondria to force protons out of the matrix and transfer to the inter-membrane space. These activities generate an electrochemical gradient which is called the mitochondrial transmembrane potential (∆Ψ). The collection of I, III, and IV units generates a proton drive force via an ATP synthase Complex V that converts adenosine diphosphate to adenosine triphosphates [16]. The transportation of proteins and carbohydrates across the inner mitochondrial membrane can influence the trans-membrane potential [17]. During the electron transport, these elements may leak and react with oxygen to produce superoxide anion that participates in the formation of reactive oxygen species (ROS). In this regard, complexes I and III are the main producers in the accumulation of ROS inside the cells, although some reports showed the potency of Complex II in ROS production [18–20]. TCA is located inside the mitochondrial matrix and is composed of a series of enzyme-catalyzed chemical reactions during aerobic respiration. All lipids, carbohydrates, or proteins contribute to the production of intermediate metabolites that incorporate with TCA to oxide carbon molecules into CO_2_ and produce energy. Most of the metabolites interact with TCA via Acetyl-CoA in distinct manners. To initiate the TCA cycle, pyruvate is transported into the matrix of mitochondria to oxidize and react with the Coenzyme A, producing Acetyl-CoA. In an alternative pathway, pyruvate can also be carboxylated to form oxaloacetate, a critical member of the TCA. During fatty acid metabolism, the β-oxidation reaction is initiated. Long-chain fatty acids are metabolized in multiple stages to ultimately constitute Acetyl-CoA. For the metabolism of proteins, glutamine is further catabolized to produce glutamate, which is by itself transformed into α-ketoglutarate [21].

In addition to the mitochondrial role on catabolic reactions, these micro-sized particles also participate in the biosynthesis of proteins, lipids, and carbohydrates. In this regard, intermediate metabolites produced by the TCA can leave the mitochondria and subsequently can be consumed as blocks for the synthesis of numerous macromolecules. It is believed that the TCA maintains its activity by procedures which are through replenishment of intermediates via a mechanism called anaplerosis [21]. Commensurate with these comments, mitochondria per se regulate the intracellular contents of amino acids and co-factors required for the functional activity of multiple enzymes, particularly DNA modifying enzymes such as histone deacetylases. The ability to maintain ionic dynamics at physiological levels could dictate the tightly regulated dynamic of ketogenesis, lipogenesis, steroidogenesis, gluconeogenesis, and ammonium detoxification [22, 23].

## The role of mitochondria in cell differentiation

Differentiation is the dynamic cellular process where an unspecific cell is committed to a specific lineage [24]. In general, differentiation encompasses changes in metabolome, genetic pattern, and proteome during a definite period. It is believed that this process is initiated from the formation of the zygote to the end of life, enabling cells to acquire specific function and phenotype [25]. Differentiation is a tightly controlled modification at the molecular levels involving methylation of specific genes, modulation of expression, and protein synthesis. Such processes need regulated energy consumption according to their adaptation to novel conditions [26, 27]. From the genetic perspective, primary and differentiated cells are the same but with different cellular functions and properties in response to extracellular signals. The existence of a steady-state and immortal energy source such as the mitochondrion could facilitate these adaptations. Not only the number and bioactivity of the mitochondria pool have a pivotal role in the promotion of differentiation, but also, the dynamics and morphology of this organelle are changed along with maturation. Other experiments also highlighted the subcellular localization of mitochondria in stem cells committed to distinct cells [28]. It has been elucidated that the maturation of myoblasts to mature myocytes after treatment with TGF-β increased respiration capacity coincided with changes in protein composition of mitochondria [29]. In this regard, the content of complex IV protein MTCO1 was significantly suppressed in the mature myocytes [29]. Noteworthy, it is well established that mitochondria are randomly scattered in the oocyte from different species meanwhile a peri-pronuclear localization of mitochondria has been observed after fertilization. Overall, not only mitochondrial number/function is critical in developmental stages but also change in subcellular clustering occurs with maturation and aging [30]. Another reason for this change would be that cell resistance to insulting conditions will be decreased by maturation due to the accumulation of oxidative stress and aged organelles occurring along with the increase of cell passage and division (Fig. 1b). To circumvent these conditions, the number of mitochondria is increased to neutralize the detrimental effects of free radicals [28, 31, 32]. A series of experiments reported that free radicals act as an antagonist to prohibit cell differentiation toward cardiovascular lineage [33, 34]. It is perhaps not surprising that mitochondrial metabolism is a regulator of the differentiation of stem cells toward cardiovascular lineage [35]. Of course, the issue becomes more complicated by the opposite result. Calling attention, it has been shown that the existence of mitochondria is beneficial to maintain the stemness feature in certain lineages. In this regard, several markers, in particular, nestin are integral to neural progenitor cells multipotentiality is affected by mitochondrial activity [36].

Odontoblastic differentiation of dental progenitor cells requires cellular activity which is related to increased extracellular mineralization, alkaline phosphatase function, and the expression of specific protein such as dentin, sialophosphoproteins, etc. The phenotype acquisition is promoted when the content of adenosine-5′-triphosphate is elevated. To afford biochemical demands, the mitochondrial potential is induced to correct the NAD^+^/NADH ratio and detoxify the free reactive oxygen ions. In support of this claim, the suppression of mitochondrial respiration by inhibitor rotenone decreases the odontogenic differentiation capacity [37]. Pouyafar and co-workers found that the inhibition of lipolysis by chemical inhibitor, namely TOFA, suppressed the endothelial differentiation and expression of VE-cadherin in human cancer stem cells [38]. Interestingly, they also found that the treatment of cancer CD133^+^ cells with Lonidamine, an inhibition of aerobic glycolysis, promoted differentiation endothelial differentiation [38]. Regarding the high basal metabolic rate and necessity for additional energy sources, it is noteworthy to mention that mitochondrial mass and ultrastructural adaptations are mandatory for efficient cell maturation and differentiation.

## Intercellular mitochondrial transfer

A body of documents stands for a fact that mitochondrial transfer actively occurs between cells from distinct types to other lineages [39]. However, elucidation of the extent, duration, and bioactivity of transferred mitochondria in the target cells needs future investigations. For example, stem cells from connective tissues known as mesenchymal stem cells (MSCs) showed a great potential to deliver and transfer mitochondria to multiple cells residing in the specific tissues in in vitro conditions and in animal models [40]. Currently, many researchers are trying to address underlying mechanisms by which these MSCs donate mitochondria to injured cells and to increase the therapeutic outcomes of stem cell therapy by the regulation of mitochondrial transfer [41]. The main hypothesis for mitochondria transfer was provided by the existence of nanotubular structures during the active mitochondrial transfer which not only harbors mitochondrial content but also the transfer of many other cellular constituents [42]. These morphological adaptations showed the unlimited potency of mammalian cells in reciprocal mitochondrial interchange in specific niches [43, 44]. Such transfer could induce numerous underlying modalities as shown previously in in vitro or in vivo conditions. Some investigations noted the active role of mitochondrial transfer in the bioactivity of cells such as lymphocytes, neurons, or cardiomyocytes. As above-mentioned, the construction of juxtacrine interaction between cells provides a secure torrent of cellular content transfer in a unidirectional or bidirectional manner.

Indeed, a panel of distinct molecules, effectors, signaling modulators, and ions participate in the formation of unique entangled molecular networks inside and between the cells. Sometimes, these contents are packed and transferred in the form of enclosed micro- and nano-sized packages such as mitochondria, lysosomes, endosomal vesicles, and other plasma membrane structures [45]. A typical example of mitochondrial transfer is referred to as the discovery of restoration of respiration capacity in mitochondria-deficient human cells [46]. Somatic cell-free mitochondria are induced by prolonged incubation with ethidium bromide and depletion of mtDNA. These cells are vulnerable and could not maintain basal metabolism in a conventional culture medium. The mitochondrial transfer can restore the aerobic respiration chain and improve cell bioactivities [46]. Liu et al. performed in vitro co-culture of MSCs with ECs under a condition similar to the ischemia–reperfusion niche. They proved the transfer and activation of MSC-derived mitochondria within the cytosol of ECs, contributing to endothelial activity in glucose- and oxygen-free medium [47]. Therefore, one could hypothesize that mitochondria transfer can promote angiogenesis and endothelial function at the site of ischemia.

In addition to an inherent capacity of stem cells to transfer mitochondrial elements to another cell type, numerous experiments showed the potency of mitochondrial transfer between immortalized cells and primary cells from the same or different species [48]. Like other cell activities, this phenomenon is associated with energy consumption and conceived as an active transfer. The formation of TNTs could be presented as the main cellular machinery of mitochondria transfer although a portion of mitochondrial structure i.e. mtDNA or mitochondrial fragments, but not whole complex, could also be transferred horizontally to target cells [48]. The main propose of mitochondrial transfer is to restore the aerobic activity of target cells with an abrogated mitochondrial activity [6, 49]. Fortunately, the mitochondrial transfer is practically happening inside the body [6, 50]. According to emerging data, attempts must be focused on the rate, quality, and intensity of mitochondrial transfer during various pathologies. Notably, certain specific condition like cancerous niche develops a unique medium to interchange a large number of mitochondria between cells residing close [6, 50]. Not as a general rule, it seems that cells displaying numerous aberrancies and injuries are more permissive to mitochondria transfer compared to healthy cell counterparts [51, 52]. To maintain mitochondrial transfer, the formation of TNTs is mandatory which has been previously proved in injured human umbilical vein ECs. In the first step, spatial arrangement, location of phosphatidylserine molecules is changed in the acceptance cells. This molecule acts as find-me signals and promotes the formation of TNTs to maintain a juxtacrine connection with surrounding cells [47]. In contrast to cells and tissues with prodigious regeneration capacity, find-me signals are more vital in tissues without cellular replacement property, in particular the central nervous system [6]. The promotion of pro-inflammatory status following bacterial lipopolysaccharide challenge was found to enable injured pulmonary epithelial cells to fuse with human stem cells by using gap junction (connexin-43), and TNTs formation [53].

Considering the complexity of mitochondrial transfer, it is reasonable to imagine that various machinery systems take part in this phenomenon. For instance, it was demonstrated that mitochondrial transfer is promoted by a close collaboration of cytoskeletal-related factors with different effectors. Among them, a protein capable of connecting mitochondria to a cytoskeletal protein termed Miro1 accelerates the transmission of mitochondria from human MSCs to asthmatic pulmonary epithelial cells [54].

Based on the evidence, there are pros and cons correlated with the mitochondrial transfer in the context of tissue niche. As previously described, it seems that mitochondrial exchange between cancer cells could induce resistance to therapeutic modalities. Even though, the mtDNA transfer between donor cancer cells and normal neighboring cells not only can change tumor dynamics but also potentiates the metabolism shift from anaerobic to the aerobic state [55, 56]. However, any modulations in the activity of the mtDNA source could regulate the dynamic growth of recipient cancer cells and may be a therapeutic approach in cancer biology.

The formation of TNTs is initiated with ultrastructural changes of the cell membrane and filopodial projections in which the use of relevant inhibitors prohibits cellular adaptation to release mitochondria. However, TNTs formation seems to be independent of the excretory capacity of cells. In line with this claim, the inhibition of phagocytosis, endocytosis, or exocytosis does not per se affect TNTs alignment in host cells although some overlapping mechanisms exist between these activities [57]. Using some approaches, we can promote the rate of mitochondrial transfer to the target cells. For instance, doxorubicin and hydrogen peroxide treatment, and expansion of cells in serum-free and high glucose conditions increase the transcription of apoptosis-related effectors such as p53 and Caspase 3. The activation of p53 promotes Caspase 3 cleavage and S100A4 degradation, participating in mitochondrial transfer and reception [58–60]. Commensurate with the above descriptions, the occurrence of pathological conditions could be touted as a stimulatory factor to circumvent mitochondria-devoid status in target cells.

### Cellular structures mediating intercellular mitochondrial transfer

Although, the exact mechanism of mitochondrial transfer is not fully elucidated different hypotheses have been considered in this regard. The most common mechanisms include membrane microvesicles, cell fusion, or mitochondrial protrusion. It seems that the modulatory effect and functional behavior of transmitted mitochondria might be dependent on physiopathological situations. Even in physiological conditions, the transmission of mitochondria is not completely safe in which some reports noted immune cell recruitment and the occurrence of mitophagy (mitochondrial death) in recipient cells after exogenous mitochondria arrival. In some cases, the mitochondrial transfer coincides with the parasite and bacterial transmission, *Entameba histolytica, Francisella tularensis,* and *Salmonella enterica* from cell to cell [61, 62].

Tumor-derived mitochondria are prone to undergo mutation and alter the cellular mechanism of recipient cells [56]. It is not surprising to implicate that mitochondria transfer from normal cells to tumor cells could retrieve a basal metabolic capacity. Previous data in this field showed that bone marrow-derived mitochondria could abrogate the accelerated angiogenesis rate during tumor development [55].

Time, mitochondrial number, and route of transfer are critical to the restoration of activity in the injured cells [63]. Therefore, the application of strategic plans is mandatory in achieving successful therapeutic effects. For instance, some researchers used the Sendai virus envelope-based approach to generate micro tunnels with length sizes ranging from 4.1 to 10 μm. Single mitochondrial transfer contributed to homoplasmy of mitochondrial DNA in the target cells. This strategy can contribute to intelligent control of mitochondrial transfer, preventing mitochondrial accumulation in recipient cells. Although cytoplasmic elongation can form cell-to-cell physical contact, it simultaneously regulates the mitochondrial flow rate to the accepting cells. Soon after mitochondrial transfer, cell connection to neighboring cells is immediately interrupted and this feature is an indicator of a successful transmission [64].

**Fig. 2:**
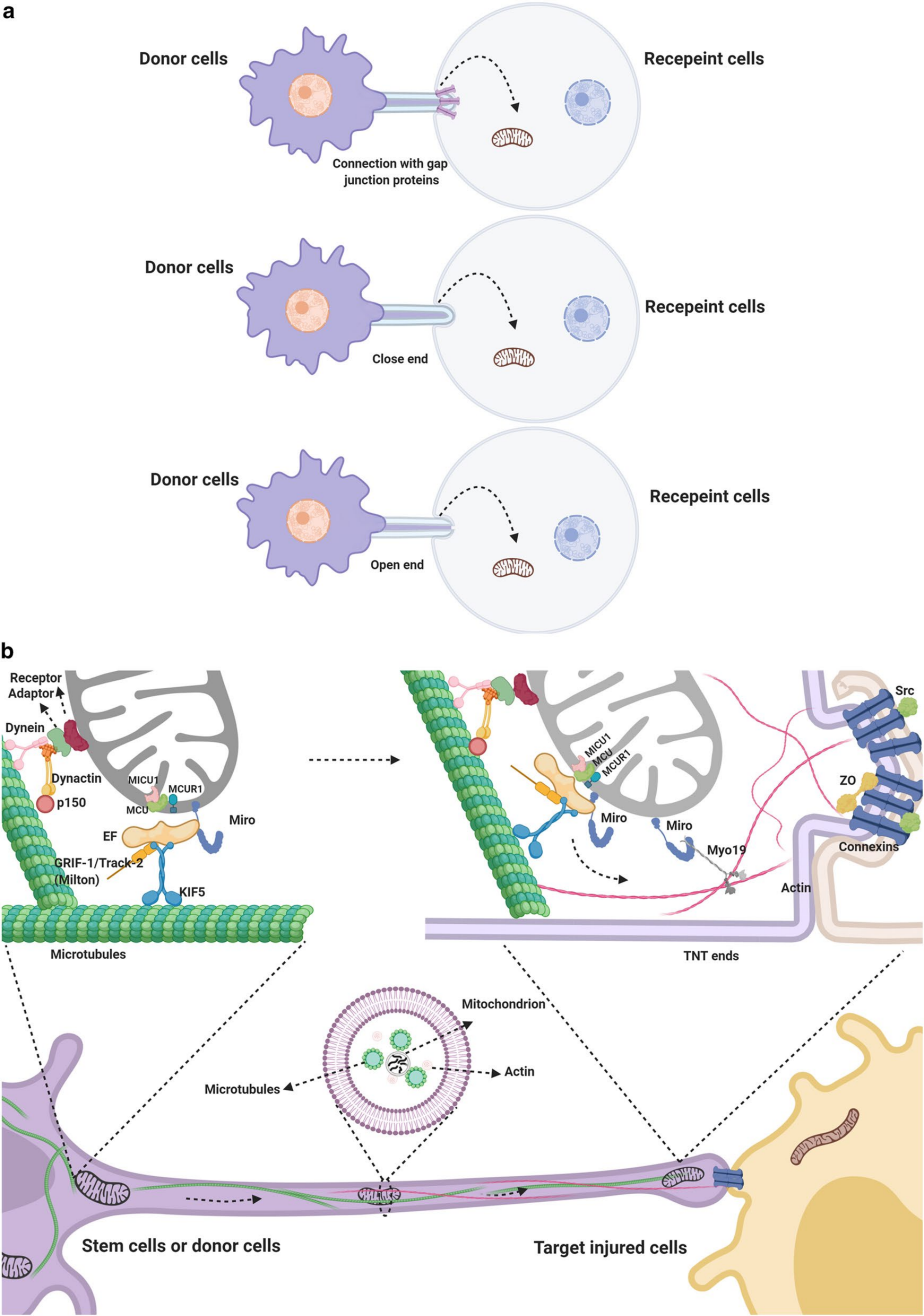
The transport of mitochondria through the TNTs formed between the donor and recipient cells (**a**). TNT bridges are close-, open-ended, or connected to the cells via gap-junction molecules. Molecular machinery participates in the transfer of mitochondria between the cells (**b**)

In general, TNTs types are based on cytoskeletal architecture, size, and function. The type 1 TNTs are characterized by long and prominent cytosolic tunnels encompassing both microfilaments and microtubules while type 2 is in small size and tubulin free. Experiments showed the capacity of type 1 TNTs in mediating organelle transfer [65, 66]. Considering the connection type, TNTs are categorized into three types namely closed, open, and gap-junction based TNTs (Fig. 2a). As above-mentioned, Miro1, mitochondrial Rho GTPase 1, is actively engaged in the mitochondrial trafficking between the cells. This factor is a calcium-dependent adaptor that closes mitochondria to the cytoskeletal scaffold (Fig. 2b) [54, 67]. In detail, Miro1 acts as an anchor to maintain the connection of mitochondria to the cell membrane and subsequently attached to the KIF5 kinesin motor. The modulation of intracellular calcium content is critical to dictate these changes. The use of calcium blockers is shown to prohibit the attachment of Miro1 to KIF5 kinesin motor. In the final stage, the binding of Miro1 to KIF5 kinesin motor initiates the formation of connexin 43-containing gap junction channels, making micro-sized tubes to orchestrate mitochondrial trafficking (Fig. 2b) [53, 68]. Interestingly, a close association of the mTOR signaling pathway with TNTs formation was previously found [58, 69]. Therefore, it could be mentioned that the activation of the mTOR axis, as seen during apoptosis and autophagy, could also potentiate the donor and recipient cells to track mitochondria reciprocally by overexpression of trafficking adaptors such [70].

By contrast, the increase of horizontal mitochondrial transfer could be used as a regenerative treatment to boost target cell metabolism. Direct injection of mitochondrial mass [71] or transplantation of MSCs [72] within the lesion zone could favor therapeutic outcomes via the mitochondrial transfer. Selecting donor cells bearing compatible connexin combinations should maximize GJC-mediated docking efficiency. Increasing mitochondrial mass by exposure to the AMP analog AICAR or a hypoxia-reoxygenation sequence would increase the number of available organelles [73]. Eventually, TNT-mediated mitochondria transfer could be stimulated either by a ROS-inducing treatment or by microtubule-stabilizing strategies (e.g., with inhibitors targeting kif11/Eg5 which acts as a ‘brake’ on microtubule extension [74], or by overexpression of trafficking adaptors such as Miro1 in the donor cells [75].

#### Mitochondrial transfer in in vitro condition

Spees et al. first demonstrated that after co-culture of lung adenocarcinoma mtDNA-deficient A549 ρ° cells with MSCs, A549 ρ° cells could uptake functional mitochondria from the MSCs [46]. Also, the isolated mitochondria from the immortalized untransformed mammary epithelial MCF-12A cells could easily enter malignant breast cancer cell lines such as MCF-7, MDA-MB-231, and NCI/ADR-Res cells compared to MCF-12A lineage. Upon mitochondrial transfer, the proliferation of these cells is suppressed in a dose-dependent pattern that coincided with increased cell sensitivity to doxorubicin, Abraxane, and carboplatin [76]. Intriguingly, vascular smooth muscle cells co-cultured with MSCs induced proliferation of MSCs through mitochondrial transfer [77].

Although it has been shown that mitochondria transfer to injured cells could restore cellular function, the release of mitochondria may result in a series of immune responses. The mitochondrial components may be recognized as damage-associated molecular patterns, which induced strong pro-inflammatory reactions in the bloodstream and extracellular medium [78, 79]. For example, mtDNA released into extracellular space induces Toll-like receptor 9-mediated inflammation and the activation of NRLP3-inflammasome [80, 81]. In a study conducted by Collins et al. [82], they found that the injection of mtDNA into mice synovial fluid resulted in severe inflammation and arthritis. However, the specific mechanisms regulating the immune system activity after mtDNA entrance to the cytosol remain unclear. Therefore, developing techniques for the intact and safe transfer of functional mitochondria to the target cells may accelerate exogenous mitochondrial donation for therapeutic purposes.

#### Mitochondrial transfer in vivo condition

Although the phenomenon of mitochondrial transfer in cell culture conditions has been widely described, it is necessary to confirm whether the mitochondrial transfer can occur in in-vivo conditions. Recently, experimental data indicated that injured neurons can capture functional mitochondria from astrocytes [83]. In this regard, CD38/CADPR/Ca^2+^ signaling may help astrocytes transfer mitochondria into neurons to promote survival and plasticity. Besides, Hayakawa et al. [6] collected extracellular mitochondria particles from primary mouse cortical astrocytes and then directly injected them into the peri-infarct cortex of mouse models of focal cerebral ischemia. After 24 h, it was found that the transplanted functional mitochondria were indeed present in neurons and cell survival signals were amplified. It seems that this process takes place during life as a dynamic process. For example, Yi et al. [84] observed mitochondrial transfer happens during embryonic development. They collected mitochondria concentrates from murine hepatocytes and then injected them into zygotes from older mice. They showed better developmental outcomes in the injected group, indicating that mitochondrial transfer can improve embryonic development. The replacement of mitochondria through nuclear transfer among oocytes has recently come into a research focus as a strategy for preventing the inheritance of mtDNA diseases [85].

Stem cells are recognized as unexceptionable donor cells for mitochondrial transfer [41]. The first evidence for mitochondrial transfer as an in-vivo therapeutic tool came from Islam et al.’s study [53]. In the sepsis acute lung injury model (airway-instilled *E. coli* LPS in anesthetized mice), BMSCs successfully transferred mitochondrial cargo to the alveolar epithelium followed by an increased ATP content and enhanced alveolar surfactant production. In such non-sterile inflammatory disease, the activation of alveolar macrophages with lipopolysaccharide makes these cells to accept mitochondria from stem cells to reduce the production of inflammatory factors. At the same time, ATP production and cell phagocytic function are stimulated [53, 86]. Lung alveolar macrophages have been shown to gain mitochondria from MSCs in both in vitro and in vivo models of acute respiratory distress syndrome resulting in the promotion of macrophage phagocytosis and improvement of bioenergetics [87]. Guo et al. [88] found that the formation of TNTs after viral infections via porcine reproductive and respiratory syndrome virus. It seems that this strategy could be effective in the transfer of stem cells-derived mitochondria to infected cells, prohibiting infected cells from apoptosis/necrosis. In sterile inflammatory diseases, stem cells are capable of alleviating the inflammatory response and rescuing injured cells [89–91]. For instance, Naji et al. indicated that the NLRP3–ASC–Caspase 1 axis induced via indium-tin-oxide nanoparticles in macrophages can provoke pyroptosis, while stem cells can inhibit the inflammatory process and rescue cardiomyoblasts from ischemia via direct cell-to-cell connections [92, 93]. Li et al. discovered that the donation of mitochondria from MSCs provides great promise for the recovery of cigarette smoke-induced lung injury in chronic obstructive pulmonary disease [40]. Notably, it is reported that there is a higher mitochondrial transfer capacity in iPSC-MSCs than that from BMSCs to repair CS-induced mitochondrial damage [94].

Mitochondria from injured somatic cells could be engulfed and degraded by stem cells which results in the induction of the cytoprotective enzyme heme oxygenase-1, an improvement of cellular proliferation and anti-apoptotic function [95, 96]. Thus, intercellular mitochondrial transfer using stem cells as a carrier holds a new approach to cure mitochondrial dysfunctional diseases [97].

## Molecular mechanism of mitochondrial transferring between cells

Organelle exchange between cells can occur via three potential means: TNTs, EVs, and cellular fusion.

### TNT-dependent mitochondria internalization mechanism

TNTs are small membranous tubes, with 50–1000 nm in diameter, seen in stem cells during mitochondrial transfer. TNTs are thin cytoplasmic extensions bordered by a plasma membrane to create a connection bridge between cells. TNTs were initially described by Rustom et al. [42] as a communicating intercellular transport network formed in the co-culture of human 293 cells and rat PC12 cells. Later, TNT formation was also reported in immune cells, including B, T, and NK cells, neutrophils, and monocytes, as well as in neurons, glial cells, prostate cancer cells, and cardiomyocytes. It is thought that TNTs are key points for effective mitochondrial transfer [57, 98]. Onfelt et al. observed that thin filaments involving F-actin and also a thicker subset (0.7 μm) containing both F-actin and microtubules participated in the formation of TNTs [99]. Meanwhile, M-sec, a mammalian protein, can induce the formation of TNTs that only contain actin filaments, but without microtubules [100]. Also, the exchange of cell particles between injured cells and stem cells was required for the formation of TNTs [47]. Also, Cdc42 (a small GTPase) plays a critical role in the TNT extension process [100]. Furthermore, the mitochondrial transfer can be induced via mitochondrial damage that releases ROS to activate NF-κB and up-regulate TNFαip2, thereby enhancing the formation of TNTs [101]. Besides, the formation of TNTs was demonstrated to be controlled by some external factors. It was observed that high concentrations of glucose play two-sided roles in the formation of TNTs. High concentrations of glucose were shown to diminish mitochondrial motility and inhibit mitochondrial trafficking in neurons via regulating Milton and its O-GlcNAcylation [102]. Moreover, TNT-mediated mitochondrial transfer from MSCs to endothelial cells was enhanced by glucose deprivation [47]. Other conditions such as low serum contents, acidic pH, exposure to H_2_O_2_, viral infection, or treatment with chemotherapeutic agents promote the formation of TNTs [50, 58, 59, 103–105]. Overall, the microenvironment around injured cells might be suited to the formation of TNTs, which is beneficial to the mitochondrial transfer. Therefore, these conditions must be defined concerning cell type and milieu. MSCs could transfer mitochondria to damaged acceptor cells via actin-based intercellular structures [106]. These cells actively transferred mitochondrial mass to NSCs and act as a shield to protect NSCs against the neurotoxic effects of cisplatin [106]. On the other hand, MSCs-to-NSCs mitochondrial transfer reversed the cisplatin-induced reduction of mitochondrial membrane potential. The inhibition of actin-based cytoskeletal re-arrangement inhibited the transfer of mitochondria to NSCs and abrogated the positive effects of MSCs on NSC survival. Conversely, overexpression of the Miro1 in MSCs increased mitochondrial transfer and further improved survival of cisplatin-treated NSCs. The activity of accessory proteins like TRAK 1 and TRAK 2 [107, 108], Myo 19, and Myo 10 [109] permit the efficient shipping of cargo between cells guided by actin–myosin-dependent mechanisms (FIG. 2a) [110]. Other experiments revealed that in vivo MSC administration prevented the loss of DCX^+^ NSCs in the subventricular zone and hippocampal dentate gyrus in animals treated with cisplatin [106]. It was shown that MSCs could donate mitochondria and protect corneal cells against oxidative stress-induced mitochondrial dysfunction. In a co-culture of MSCs and CECs, the mitochondrial transfer was enhanced from MSCs to CECs after treatment with Rotenone (Rot)-induced oxidative stress [101]. Separation of MSCs and CECs by a Transwell® culture system aborted mitochondrial transfer from MSCs to CECs. Mechanistically, increased filopodia outgrowth in CECs for TNT formation was associated with oxidative inflammation-activated NF-*κ*B/TNF*α*ip2 signaling pathways that could be attenuated by reactive oxygen species scavenger *N*-acetyl cysteine treatment. Furthermore, MSCs grown on a decellularized porcine corneal scaffold were transplanted onto an alkali-injured eye in a rabbit model [101]. Enhanced corneal wound healing was evident following healthy MSC scaffold transplantation and transferred mitochondria were detected in corneal epithelium. Mitochondrial transfer from MSCs provides novel protection for the cornea against oxidative stress-induced mitochondrial damage [101]. The balance between mitochondrial fusion and fission events pre-determines the morphological adaption of this organelle in target cells. Even, the precise and successful cross-talk between de novo mitochondrial source with host cell mitochondrial pool and other organelles ensues efficient proteomic and genomic modulation and participation of mtDNA in favor of cell bioactivities [111, 112]. However, it is a naïve idea to imagine that mitochondria transfer is commonly accepted by the target cells and in most cases, the cell-based mitochondrial activity could not be restored even after mitochondrial transport [46].

### Cell fusion

**Fig. 3:**
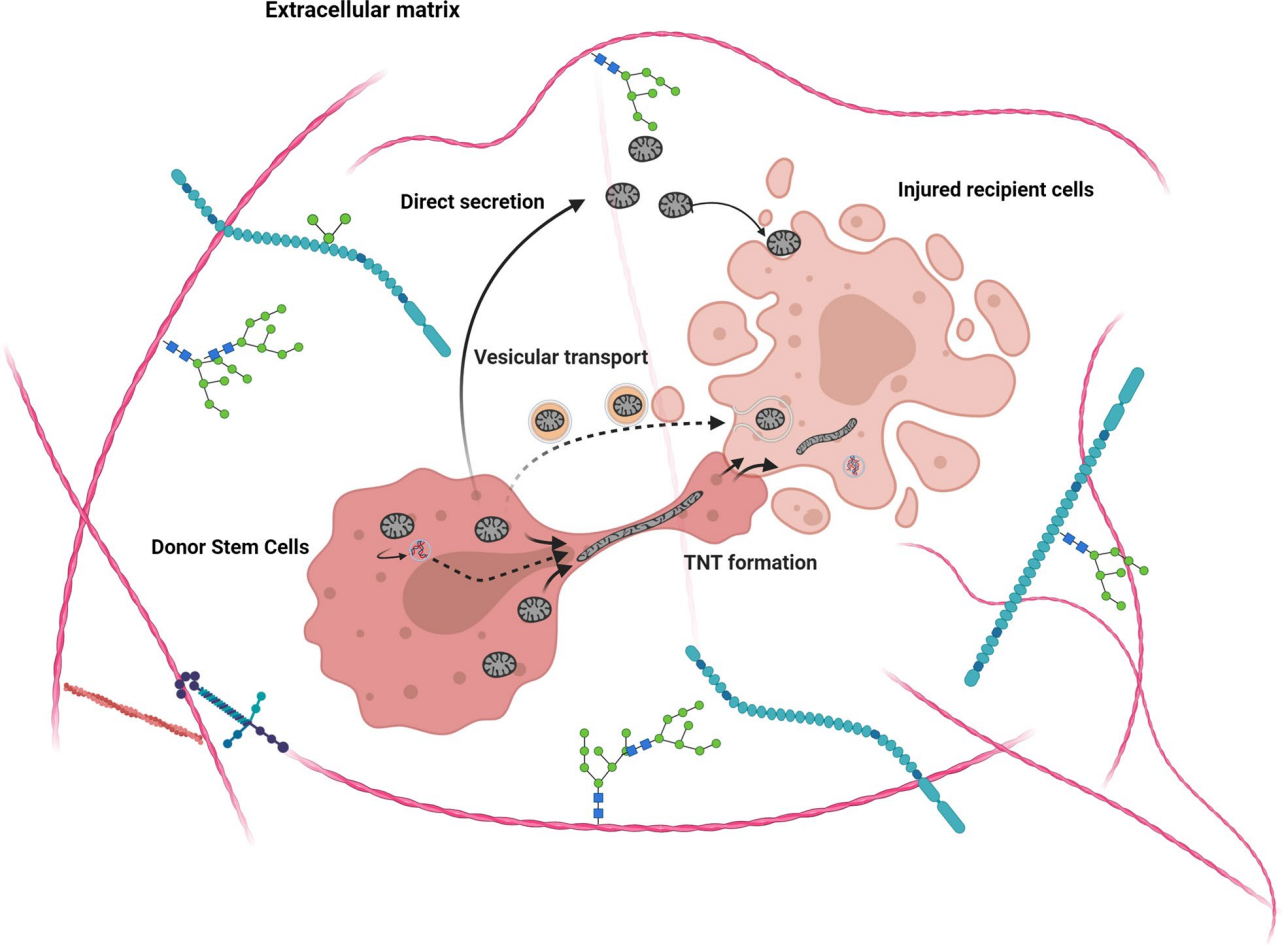
Different pathways are available for mitochondrial transfer between the cells. The common method for mitochondria transfer is done via TNT bridges while mitochondrial fragments or products, mtDNA, could be transferred via extracellular vesicles. In some cases, the mitochondrial mass is directly engulfed by the acceptor cells

Cell fusion is another mechanism by which cells are interconnected physically by the plasma membrane of two distinct cell types with retained nuclear morphology. Under these situations, cytosolic constituents and organelles are evenly shared between juxtaposed cells with a permanent fusion. Although, partial cell fusion could also exchange subcellular organelles, like mitochondria, and protein complexes, this kind of fusion is rare and happens under specific circumstances [113]. Similar to vesicular fusion and secretion, the mitochondrial transfer is started since the mitochondria outer membrane has physical contact with the plasma membrane. Similar to the formation of TNTs for mitochondrial distribution, the majority of molecular pathways are the same as the above-mentioned (Fig. 3) [114].

## Role of extracellular vesicles in directing the intercellular mitochondrial transfer

Organelle transfer is actively seen between cells via the secretion of micro and nano-sized EVs. EVs are found in numerous in vivo samples such as urine, plasma, etc. Based on the size and route of biogenesis, EVs are classified into three main categories: exosomes, microvesicles, and apoptotic bodies [63, 115]. Exosomes are small homogenous membrane-coated vesicles ranging from 30 to 100 nm in diameter [116, 117]. Upon reaching the target cells, EVs are internalized and release their cargo to acceptor cells. Therefore, EVs act as messengers for long- and short-distance crosstalk among the cells [118–120]. Due to their small size, it is unlikely that microvesicles and nano-vesicles such as exosomes (ranging from 40 to 200 nm) carry mitochondria, but these elements are touted as suitable bio-shuttles to transfer the factors that facilitate the mitochondrial transfer between the cells (Fig. 3). Increasing evidence showed that exosomes are eligible mediators for harboring mitochondrial genome to the recipient cells [121]. Most notably, mtDNA-laden exosomes could be considered as an alternative method to transfer mitochondrial associated bioactivity to specific cells in addition to direct mitochondrial transfer. The ability of exosomes and EVs to transfer other genetic elements that are potent to regulate mitochondrial activity inspires the fact that these vesicles could indirectly affect the mitochondrial related activity in the candidate cells. Since the interaction of exosomes with the target cells is governed by different exosome-cell interactions, it is mighty to imagine that the induction of protective effects via mitochondrial genome hindered by exosomes in a paracrine changing the metabolism of multiple cells at the same time. In this regard, Islam et al. reported the term EV-mediated mitochondrial transfer in MSCs towards alveolar epithelial cells [122]. Other experiments done by Phinney et al. confirmed that MSC EVs could transfer functional mitochondria that were successfully internalized by macrophages contributed to activated oxidative phosphorylation. Irrespective of the fact that mitochondria could shed protective effects by exosomes cargo or EVs indirectly regulates mitochondrial activity in the target cells by other elements, both implications contribute to inspiration of definite implication that exosomes and/or EVs are active or passive modulators of mitochondrial activity in the target cells. Due to size limitation, it is an illusion to conceive that exosomes could carry mitochondrial mass between cells while large-sized EVs such as apoptotic bodies (ranging from 500 to 1,000 nm) are mighty eligible to carry mitochondria which have comparable sizes [123]. In support of this claim, cells undergoing apoptotic changes could be touted as a mitochondrial exporter to juxtaposed or remote cells. As above-mentioned in this article, cells with injuries are at the center of attention and the pro-inflammatory condition makes these cells to affect the activity of mitochondria in other cells. In conclusion, due to the dynamic state of cells under pathological and physiological conditions, it is not unlikely to say that each cell could be introduced as mitochondrial exporter and/or receiver depended on the milieu and energy demands.

### Mitochondrial transfer in the field of cardiovascular disease

It has been well-documented that MSCs could release an array of soluble factors like growth factors, cytokines, and chemokines [124]. According to mechanisms of action, different reparative aspects are affected soon after the transplantation of MSCs into injured cardiac tissue. These cells could increase capillary density (angiogenesis), and regulate cardiac contractility, fibrosis, and remodeling [1, 125]. Besides, the number of apoptotic cardiomyocytes is reduced via the secretion of factors such as VEGF, FGF-2, HGF, IGF-I, leading to enhanced cardiac function [126]. The secretion of specific cytokines, PGE_2_, IL-10, etc. could help the local cardiomyocytes to protect themselves from subsequent inflammatory response cascades [124, 127]. Commensurate with these comments, a plethora of documents have shown that paracrine activity and regulation of inflammatory responses are the main underlying mechanisms driven by MSCs rather than in situ proliferation and differentiation toward cardiomyocyte-like cells [128]. Regarding the high intracellular mitochondrial content in the cardiovascular system, it is logical to mention that the transfer of mitochondria to vascular and cardiac cell lineages reveals potentially therapeutic effects. The transfer of mitochondria from MSCs or other stem cell types to cardiomyocytes and different adult cells was confirmed previously [6, 129] (Table 1). It is worth mentioning that the accumulation of pro-inflammatory cytokines such as TNF-α, IL-6, etc. at the site of injury could induce massive cytoskeletal re-arrangement in transplant stem cells, leading to facilitation in the formation of TNTs and mitochondrial transfer to neighbor cells. Recently, inflammation-associated mitochondrial donation of MSCs to different cell lineages such as cancer cells, retinal cells, and pulmonary epithelial cells has been proved [130]. These features show that the regenerative effects of stem cells and the extent and direction of mitochondrial transfer highly depend on a microenvironment. Calling attention, a pro-inflammatory environment is an incentive factor to maintain MSC-derived mitochondrial transfer immune cells such as T lymphocytes which per se regulate host immune cells activity [131]. Along with these comments, it seems that there is a close association between MSCs paracrine activity and mitochondrial donation to promote regeneration of cardiac tissue. Of course, it should be noted that the severity of inflammatory responses should not be such that disrupt the potential ability of MSCs to transfer mitochondria to acceptor cells. Considering the promising cardioprotective outcomes after mitochondria transplantation in different animal models, the establishment of the first clinical trial in pediatrics myocardial ischemia–reperfusion injury paved a way to cure patients with cardiac insufficiencies [132]. Although the ability of source cells, such as MSCs, have been confirmed in the formation of TNTs for mitochondrial distribution, it seems that the use of appropriate cells could yield therapeutic outcomes [133]. In contrast to fibroblasts with limited mitochondrial supply, ex vivo modulation of MSCs potentiates these cells to form TNTs for better mitochondrial transfer [134]. The direct or indirect injection of exogenous mitochondria showed a fast-cellular internalization of these organelles by various cardiac resident cells such as cardiomyocytes and fibroblasts by the restoration of mtDNA and energy demands [135, 136]. The cross-talk between cardiac and non-cardiac cells pre-determines the efficiency of mitochondrial therapy [137, 138]. The route of mitochondrial introduction is considered as a critical step in the restoration of the cardiac outcome. Intracoronary infusion of mitochondria yielded in the fast and efficient distribution of mitochondria throughout the whole cardiac tissue. This strategy enables us to use a high therapeutic dose of mitochondrial therapy as was previously used in Langendorff-perfused rabbit infarcted cardiac tissue [135]. The engagement of Caveolae-dependent-clathrin dependent endocytosis, actin-meditated endocytosis, TNTs formation, actin-mediated endocytosis, and macro-pinocytosis is thought to be underlying mechanisms in mitochondrial therapy of cardiac insufficiencies [59, 139, 140]. The lack of mitochondrion-lysosome fusion in target cardiomyocytes revealed the exploitation of anti-phagocytic mechanisms to exclude newly entered mitochondria [141].

**Table 1:** Transfer of mitochondrial donation in different in vivo and in vitro systems

Milieu	Donor cells	Recipient cells	Outcome	References
In vitro	Human bone marrow MSCs	Adult mouse cardiomyocytes	Mitochondrial transfer is required for somatic cell reprogramming	[129]
			Heterologous cell fusion promoted cardiomyocyte reprogramming back to a progenitor-like state.	
In vivo	Human induced-pluripotent-stem-cell-derived MSCs (iPSC-MSCs)	Cardiomyocytes	iPSC-MSCs has superior effect to transfer mitochondria due to enhanced expression of Miro-1	[72]
			The higher levels of TNFαIP2 expression in iPSC-MSCs make them respond to TNF-α-induced TNT formation to transfer mitochondria to anthracycline-induced cardiomyocytes.	
			Suppression of TNFαIP2 or MIRO1 in iPSC-MSCs aborted mitochondrial transfer.	
In vitro	Human MSCs	Rat cardiomyocytes	The co-culture of rat cardiomyocytes with human MSCs increased the number of TNTs.	[142]
In vitro and in vivo	Rabbit fibroblast isolated from cardiac tissue	Adult rabbits cardiomyocytes	An inter-cytoplasmic connection is provided between fibroblasts and dedifferentiated cardiomyocytes.	[143]
			Disruption of the basal lamina was initiated after TNT formation in the border zone of a rabbit myocardial infarction.	
In vitro	Normal mouse MSCs	Ischemic H9C2 cardiomyoblasts	Wide (200–500 nm) intercellular connections formed between the rat cardiomyoblasts and mouse MSCs	[93]
			Cell fusion rarely occurred between the rat cardiomyoblasts and mouse MSCs.	
In vitro	Rat MSCs	Rat neonatal cardiomyocytes	MSCs make cell-to-cell connection by initial extension of filopodia.	[134]
			Unidirectional transfer of mitochondria occurred between MSCs and cardiomyocytes.	
			Compared to the MSCs, few TNT formations were observed between the cardiac fibroblasts and cardiomyocytes in a homotypic or mixed cell population.	
In vivo	Mouse astrocytes	Mouse neurons	CD38 and cyclic ADP ribose signaling participate in mitochondrial transfer	[6]
In vitro	Bone marrow MSCs	Rat renal tubular cells	The transport of cellular components was started three hours after co-culturing	[144]
			Both anterograde and retrograde mitochondrial transfer were seen between the MSCs and renal tubular cells.	
			Renal-specific Tamm-Horsfall protein was induced in MSCs after connection to the renal cells, promoting MSCs differentiation toward tubular cells.	
In vitro	Adult human endothelial progenitor cells	Rat cardiomyocytes	The number endothelial progenitor cell-derived TNTs increased six hours after co-culturing.	[133]
			Transport of MitoTracker-positive structures was done from cardiomyocyte toward endothelial progenitor cells.	
			The acquisition of a cardiomyogenic phenotype was recorded in endothelial progenitor cells independent of cellular or nuclear fusion.	
In vitro	Human bone marrow MSCs	Human umbilical vein endothelial cells (HUVECs)	TNT-like structure was performed between MSCs and HUVECs.	[47]
			Oxygen/glucose deprivation and re-oxygenation in HUVECs induced unidirectional mitochondrial transfer through TNTs from MSCs.	
			Formation of TNTs is a defense and rescue mechanism after exposure of phosphatidylserine on the surface of apoptotic endothelial cells.	
In vitro and in vivo	Neonatal rat cardiomyocytes	Neonatal rat cardiomyocytes	Mitochondrial internalization is done through actin-dependent endocytosis.	[136]
			Internalized mitochondria replenished cardiomyocyte ATP content.	
			Oxygen consumption increased after mitochondrial internalization.	
In vitro and in vivo	Cardiac fibroblasts	Myocytes	In response to cardiac injury, interactions between myofibroblasts and myocytes are enhanced, contributing to significant electrophysiological changes and influencing electrotonic connectivity between cardiomyocytes and fibroblasts and/or myofibroblasts	[145]
In vitro	Human uterine endometrial gland MSCs	Rat H9C2 cardiomyoblasts	Mitochondrial transfer was seen in homogeneic and xenogeneic cells.	[140]
			Mitochondrial transfer rescued the mitochondrial respiratory function and improved the cellular viability in mitochondrial DNA-depleted cells.	
			Micropinocytosis participates in mitochondrial internalization.	
In vitro	Rat MSCs	Neonatal cardiomyocytes	Connexin-43 was induced as junctional factors between the MSCs and cardiomyocytes.	[146]
			MSC-cardiomyocyte fusion was initiated.	
			Partial cell fusion and TNT accelerated the transfer of MSC mitochondria to the cardiomyocytes.	
In vitro and in vivo	Rat cardiac fibroblasts	Neonatal rat cardiomyocytes	Microtubules and motor protein KIF5B are required for mitochondrial transport from fibroblasts to cardiomyocytes.	[147]
			The mitochondrial transfer was observed from fibroblast to hypoxia-treated cardiomyocytes but not vice versa	
			Intact and hypoxia/re-oxygenation-treated fibroblast decreased cardiomyocyte apoptosis by mitochondrial donation via TNTs.	
In vitro and in vivo	Human-induced pluripotent stem cell (iPSC)-derived MSCs	Asthmatic epithelial cells	iPSC-MSC transplantation decreased T helper 2 related cytokines and blunted mitochondrial dysfunction in epithelial cells	[148]
			TNTs were formed between iPSC-MSCs and epithelial cells	
			Mitochondrial transfer was done from iPSC-MSCs to epithelial cells via TNTs	
			Connexin 43 plays a critical role in the regulation of TNT formation in iPSC-MSCs.	

## Conclusion

It seems that mitochondrial transfer could be touted as a novel and efficient approach in which stem cells could exert therapeutic outcomes in the injured tissues. Due to the complexity of the mechanisms involved in mitochondrial donation, the impact of different factors such as the impact of type and intensity damage in the ability of cells to obtain mitochondria, the potential capacity of mitochondrial donor cells, alternative routes participating in mitochondrial transfer should be addressed in the future studies.

## Data Availability

Not applicable.

## Abbreviations

BMSCs: Bone marrow mesenchymal stem cells
CECs: Corneal epithelial cells
EVs: Extracellular vesicles
FGF: Fibroblast growth factor
HGF: Hepatocyte growth factor
IGF-I: Insulin-like growth factor-I
IL-10: Interleukin 10
Miro1: Mitochondrial motor protein Rho-GTPase 1
NSCs: Neural stem cells
PGE2: Prostaglandin E2
TNFα: Tumor necrosis factor-α
TNTs: Tunneling nanotubes
VEGF: Vascular endothelial growth factor
